# In vivo Fitness of *Acinetobacter baumannii* Strains in Murine Infection Is Associated with International Lineage II-rep-2 and International Lineage III Clones Showing High Case Fatality Rates in Human Infections

**DOI:** 10.3390/microorganisms8060847

**Published:** 2020-06-04

**Authors:** Amir Nutman, Jonathan Lellouche, Ziv Lifshitz, Rivka Glick, Yehuda Carmeli

**Affiliations:** 1 National Institute for Infection Control and Antibiotic Resistance, Tel-Aviv Sourasky Medical Center, 6423906 Tel-Aviv, Israel; jonathanl@tlvmc.gov.il (J.L.); zivlif@gmail.com (Z.L.); rivitu@gmail.com (R.G.); yehudac@tlvmc.gov.il (Y.C.); 2 Sackler Faculty of Medicine, Tel-Aviv University, 6997801 Tel-Aviv, Israel

**Keywords:** carbepenem-resistant *Acinetobacter baumannii* (CRAB), fitness, in vitro growth curve assay, in vivo murine infection model, outcome, 14-day mortality, case fatality rate

## Abstract

We previously reported that the 14-day case fatality rate (CFR) in patients with carbapenem-resistant *Acinetobacter baumannii* (CRAB) bacteremia varied between infecting clones. Here, we evaluated the in vitro and in vivo fitness of CRAB blood isolates belonging to clones with low CFR (< 32% 14-day mortality) and high CFR (65% 14-day mortality). Fitness was measured in vitro using a growth curve assay and in vivo using murine thigh muscle and septicemia models of infection. Our sample included 38 CRAB isolates belonging to two clones with low CFR (international lineage (IL)-II-rep-1, n = 13 and IL-79, n = 6) and two clones with high CFR (IL-III, n = 9 and IL-II-rep-2, n = 10). In in vitro growth curves, mean lag time, generation time and maximal growth varied between clones but could not discriminate between the high and low CFR clones. In the in vivo models, bacterial burdens were higher in mice infected with high CFR clones than in those infected with low CFR clones: in thigh muscle, 8.78 ± 0.25 vs. 7.53 ± 0.25 log_10_CFU/g, *p* < 0.001; in infected spleen, 5.53 ± 0.38 vs. 3.71 ± 0.35 log_10_CFU/g, *p* < 0.001. The thigh muscle and septicemia model results were closely correlated (r = 0.93, *p* < 0.01). These results suggest that in vivo but not in vitro fitness is associated with high CFR clones.

## 1. Introduction

*Acinetobacter baumannii* is an important nosocomial pathogen [1]. *A. baumannii* strains resistant to virtually all classes of antibiotics have emerged, with limited treatment options [2]. In a recent study, 14-day mortality among patients with severe infections caused by carbapenem-resistant *A. baumannii* (CRAB) was 37% [3]. A meta-analysis of studies comparing patients with carbapenem-susceptible and carbapenem-resistant *A. baumannii* infections found that the latter had 2.5-fold higher odds of death [4]. Bacteria with acquired resistance mechanisms often have reduced fitness; however, compensatory mutations may allow these bacteria to adapt and regain fitness and virulence [5,6]. The measurement of planktonic growth rates offers a good model for evaluating the fitness of bacterial strains [6]. However, behavior in in vitro conditions does not necessarily correlate with virulence [5,7]. Animal models have been widely accepted for the prediction of bacterial pathogenicity. Common murine models include pneumonia [8,9], skin and soft tissue infection [10,11], sepsis [12,13] and thigh muscle infection [14,15,16].

Molecular typing methods are needed for epidemiologic investigations of CRAB hospital outbreaks and surveillance of CRAB isolates [1]. Several genotyping methods exist, however, multi-locus sequence typing (MLST) has become the gold-standard [17]. Single-locus sequence typing (SLST) of the *bla*_OXA-51-like_ is a simple and cheap method for CRAB identification and typing, with good correlation with MLST, and has been successfully used to assign CRAB isolates to international clonal lineages (IL) [18]. In a previous study, we found an association between the CRAB clonal group and the 14-day case fatality rate (CFR) among patients with CRAB bacteremia. CFR ranged from 17% to 65% depending on the clonal group [19]. Here, we aimed to evaluate the association between CRAB in vitro and in vivo fitness and clonal groups with high and low CFR in patients with CRAB bacteremia.

## 2. Materials and Methods

### 2.1. Bacterial Isolates

CRAB isolates were collected from hospitalized patients with CRAB bacteremia at Tel-Aviv Sourasky Medical Center, Israel, between 2008 and 2011 as part of a previously published case–control study [19]. Microbiological methods have been described previously [19]. In brief, all isolates were identified at the *Acinetobacter baumannii*–*calcoaceticus* complex level by VITEK^®^ 2 (bioMérieux SA, Marcy l’Etoile, France). Genomic species identification of *A. baumannii* was performed by the presence of the *bla*_OXA-51-like_ gene and allelic determination using *bla*_OXA-51-like_ gene sequencing [18]. Resistance to carbapenems and antibiotic susceptibility was determined by VITEK^®^ 2 (bioMérieux SA, Marcy l’Etoile, France) and interpreted according to the Clinical and Laboratory Standards Institute (CLSI) guidelines [20]. To determine clonal relatedness among the CRAB isolates, we used two molecular typing methods, as described previously [19]. First, we performed single-locus sequence typing (SLST) of the *bla*_OXA-51-like_ gene to assign isolates to international clonal lineages (IL) [18]. In addition, we used repetitive extragenic palindromic PCR (rep-PCR) typing to reflect the more recent clonal divergence of the isolates [21,22]. A clonal group was defined according to IL and ≥80% similarity by rep-PCR typing among its members [19]. CFR was defined according to 14-day all-cause mortality. Thirty-eight isolates representing four different clonal groups were selected for this study: 19 belonging to two low CFR clones (< 32% mortality) and 19 belonging to two high CFR clones (65% mortality). The low CFR clones were IL-II-rep-1 (n = 13) and IL-79 (n = 6), and the high CFR clones were IL-II-rep-2 (n = 10), and IL-III (n = 9). A list of included isolates with minimum inhibitory concentrations (MIC) is presented in Supplementary Table S1.

### 2.2. Growth Curve Assay

Growth curve assay was performed for 36 isolates: IL-II-rep-1 (n = 13), IL-79 (n = 6), IL-III (n = 8) and IL-II-rep-2 (n = 9). Frozen isolates were sub-cultured in 20 mL Luria–Bertani (LB) broth (Hylabs, Rehovot, Israel) and incubated overnight (18–24h) at 37 °C with shaking. Fresh overnight growths of bacteria were diluted to produce the desired starting inoculum (approximately 10^2^ colony forming units (CFU)/mL) and aliquots with a volume of 200 µL were transferred to 96-well microplates (CELLSTAR, Greiner Bio-One, Austria). The plates were incubated aerobically for 24h at 37°C with shaking. Cell growth was followed by measuring the optical density at a wavelength of 600 nm (OD_600_) using a microplate reader (Synergy 2, BioTek Instruments, USA). OD_600_ values were read every 10 minutes and data were recorded using microplate data collection and analysis software (Gen5, BioTek, USA). Lag time, generation time and maximal growth (OD_600-max_) were estimated by fitting the modified Gompertz equation [23]. Four experiments were performed on each isolate and the results were averaged.

### 2.3. Murine Infection Models

We used two murine infection models in to test the in vivo growth of the study isolates. The primary model was the thigh muscle infection model [14,15]. A sub-sample of isolates was also tested in the sepsis model [12,13], which models a systemic infection. In both models, the in vivo fitness of an isolate was defined as the CFU count 24 h after injection of an initial inoculum of 10^5^ CFU.

### 2.4. Thigh Muscle Model

Thigh muscle infection was performed for 33 isolates: IL-II-rep-1 (n = 11), IL-79 (n = 5), IL-III (n = 9) and IL-II-rep-2 (n = 8). Female ICR (CD1) mice (Envigo, Jerusalem, Israel) between 5 and 6 weeks of age were acclimatized with free access to food and water for one week prior to infection. Mice were rendered neutropenic by intraperitoneal injections of cyclophosphamide (Sigma, St Louis, Missouri) at concentrations of 150 and 100 mg/kg of body weight, given 4 days and 1 day prior to infection. Cultures were prepared as described above. On the day of infection, 0.1 mL of bacterial culture (approximately 10^5^ CFU) was injected intramuscularly to the left thigh. To determine bacterial burdens, mice were euthanized after 24 h and thighs were aseptically removed and homogenized individually in 1 mL of saline solution. Serial dilutions were plated on Mueller–Hinton (MH) agar plates (Hylabs, Rehovot, Israel) and incubated overnight at 37 °C. Based on preliminary studies which showed high repeatability of bacterial counts at 24 h, two experiments were performed for each isolate, and the results were averaged. Results were presented as the number of CFU per gram of thigh muscle.

### 2.5. Sepsis Model

Twelve randomly selected CRAB isolates were further evaluated in the sepsis model: IL-II-rep-1 (n = 4), IL-79 (n = 2), IL-III (n = 3) and IL-II-rep-2 (n = 3). The protocol was the same as for the thigh model except that the bacterial inoculum was injected intravenously into the tail vein and the bacterial burden was measured in the spleen (CFU/g spleen).

### 2.6. Statistical Analysis

The fitness of a clonal group was defined as the average growth (in the in vivo and in vitro models) of isolates belonging to the group. Results were presented as mean ± standard deviation (SD). The association between fitness and clonal group was tested by an ANOVA. To compare low and high CFR clones, we pooled the results of the two low CFR clones and the results of the two high CFR clones, and tested the difference in fitness between the two groups by a t-test. The correlation between the results of the murine models was tested by Pearson’s correlation. Statistical significance was set at *p* <0.05. Data analysis was performed using SPSS version 25 (IBM Corporation, Armonk, NY).

### 2.7. Ethics

The study protocol was approved by the Tel-Aviv Sourasky Medical Center Institutional Review Board and Animal Care and Use Committee.

## 3. Results

### 3.1. Growth Curve Assay

**Figure 1:**
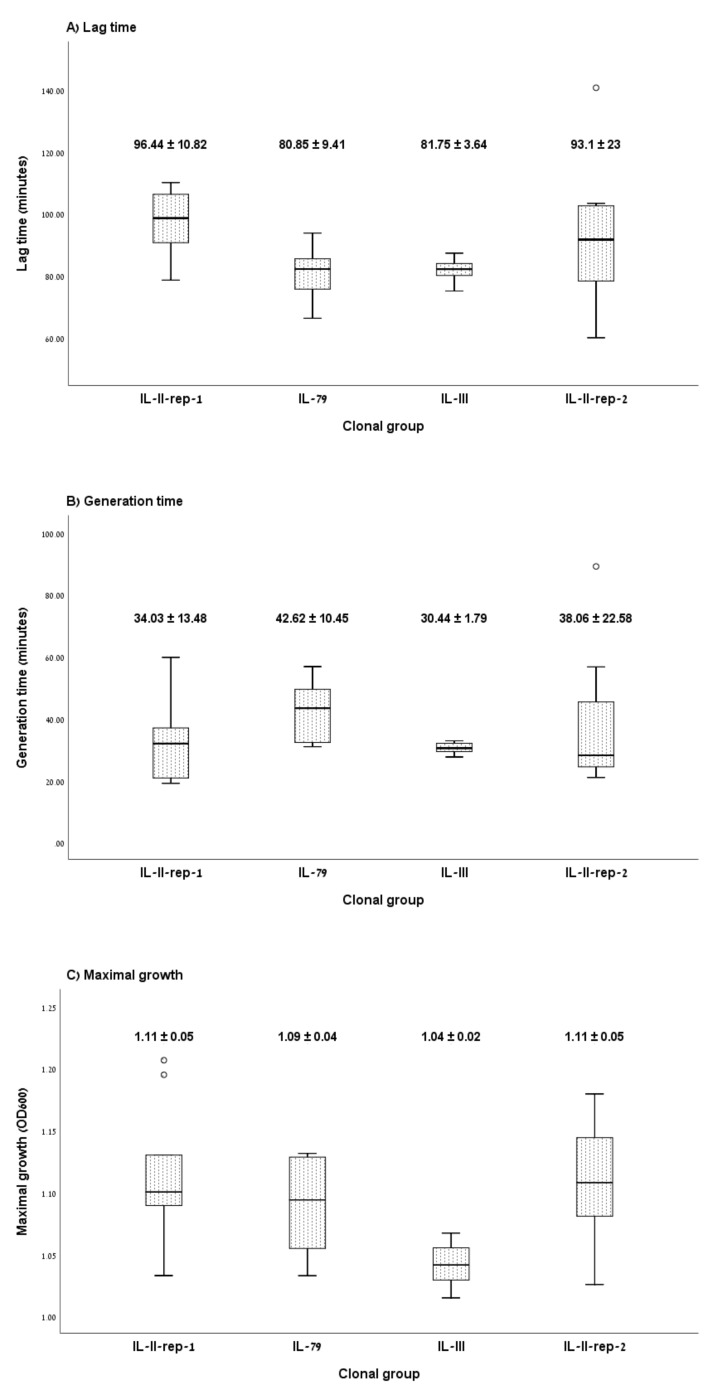
Effect of clonal group on in vitro fitness. (**A**) lag time, (**B**) generation time and (**C**) maximal growth after overnight incubation of isolates belonging to the carbapenem-resistant *Acinetobacter baumannii* (CRAB) clonal groups with low case fatality (IL-II-rep-1 and IL-79) and high case fatality (IL-III and IL-II-rep-2). Box plots depict the median (central horizontal lines), inter-quartile range (boxes) and minimal/maximal values (whiskers). Numerical values are the mean and standard deviation.

Figure 1 presents the planktonic growth curve characteristics by clonal group. The mean lag time was 96.44 ± 10.82 min for IL-II-rep-1, 80.85 ± 9.41 min for IL-79, 81.75 ± 3.64 min for IL-III and 93.1 ± 23 min for IL-II-rep-2 (*p* = 0.051) (Figure 1A). Pooled together, the difference in lag time between the high and low CFR clones was not statistically significant (*p* = 0.47). The mean generation time did not differ significantly between the four clones: 34.03 ± 13.48 min for IL-II-rep-1, 42.62 ± 10.45 min for IL-79, 30.44 ± 1.79 min for IL-III and 38.06 ± 22.58 min for IL-II-rep-2 (*p* = 0.44) (Figure 1B). The maximal growth (OD_600-max_) varied between clones: 1.11 ± 0.05 for both IL-II-rep-1 and IL-II-rep-2, 1.09 ± 0.04 for IL-79 and 1.04 ± 0.02 for IL-III (*p* = 0.003) (Figure 1C); however, the difference between the high and low CFR clones was not statistically significant (*p* = 0.12). Growth curve results for each isolate are presented in Supplementary Table S2.

### 3.2. Murine Infection Models

**Figure 2:**
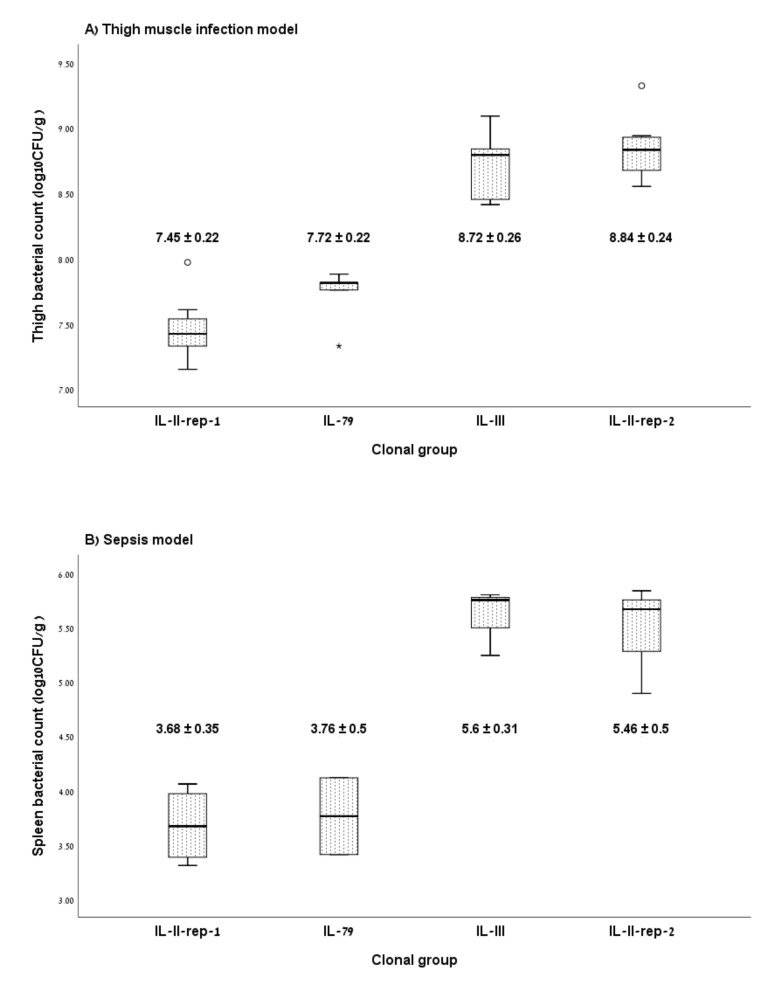
Effect of clonal group on in vivo fitness. Bacterial counts in thigh muscle (**A**) and spleen (**B**) following infection with isolates belonging to CRAB clonal groups with low case fatality (IL-II-rep-1 and IL-79) and high case fatality (IL-III and IL-II-rep-2). Box plots depict the median (central horizontal lines), inter-quartile range (boxes) and minimal/maximal values (whiskers). Numerical values are the mean and standard deviation.

Figure 2 shows the mean bacterial counts recovered from infected mice. In the thigh muscle model (Figure 2A), the mean bacterial counts were 7.45 ± 0.22 log_10_CFU/g for IL-II-rep-1, 7.72 ± 0.22 log_10_CFU/g for IL-79, 8.72 ± 0.26 log_10_CFU/g for IL-III and 8.84 ± 0.24 log_10_CFU/g for IL-II-rep-2 (*p* < 0.001). Bacterial counts were significantly higher for the two high CFR clones as compared with the two low CFR clones (8.78 ± 0.25 log_10_CFU/g vs. 7.53 ± 0.25 log_10_CFU/g, *p* < 0.001).

In the sepsis model (Figure 2B), the mean bacterial counts were 3.68 ± 0.35 log_10_CFU/g for IL-II-rep-1, 3.76 ± 0.5 log_10_CFU/g for IL-79, 5.6 ± 0.31 log_10_CFU/g for IL-III and 5.46 ± 0.50 log_10_CFU/g for IL-II-rep-2. Bacterial counts were significantly higher for the two high CFR clones as compared with the two low CFR clones (5.53 ± 0.38 log_10_CFU/g vs. 3.71 ± 0.35 log_10_CFU/g, *p* < 0.001). The bacterial burden in the thigh model and in the sepsis model were closely correlated (r = 0.93, *p* < 0.001). Results for each isolate are presented in Supplementary Table S2.

## 4. Discussion

In this study, we examined the relation between in vitro and in vivo fitness models and clonal groups associated with mortality in patients with CRAB bacteremia [19]. In the first approach, we evaluated fitness using an in vitro planktonic growth curve assay. In vitro characteristics showed clonal variations in lag time and maximal growth rate; however, they did not discriminate between the high and low CFR clones. These results highlight the limitations of the in vitro assays to predict clinical outcomes. We also evaluated the fitness of CRAB isolates using two different in vivo murine models of infection. In contrast to the in vitro data, in the murine models, the bacterial burden in the infected organ correlated with the CFR of the clones. Organ bacterial counts in the thigh and spleen models were closely correlated.

While in vitro assays are easier to perform, and ethically preferable, studies have shown a lack of correlation between in vitro fitness and virulence [24,25]. Thus, in vitro results should be interpreted with caution. In vivo models are needed to assess virulence and define virulence factors. These models must be relevant to human infection. The limitation of many animal models is that it is unclear how well they correlate with clinical outcome, since routes of infection are often non-physiologic (for instance, intra-peritoneal injection), or mice are rendered immunocompromised [26]. Our study provides evidence that murine infection models can be used to stratify CRAB clonal groups according to risk. The utility of the thigh muscle model is its ease and reproducibility compared with other models.

Our study has a number of limitations. It was a single-center study; therefore, results may not be generalizable to all settings. As the current study was a follow-up study, we used the same clonal grouping methods for the sake of consistency. Thus, our study did not provide data on the phylogenetic relationship between clones, and therefore cannot provide evidence on the evolution of fitness. We did not characterize virulence factors. Furthermore, we could not evaluate mortality in the murine models, since all mice were euthanized after 24h to determine the bacterial burden. Studies should also examine the correlation between murine models of CRAB infection and lower class in in vivo models such as *Galleria mellonella* (a moth) and *Caenorhabditis elegans* (a nematode), which have been shown to correlate with mammalian models for their ability to discriminate between degrees of virulence [27,28,29].

In conclusion, in vitro growth varied between CRAB clones but was not associated with case fatality. The in vivo fitness in murine infection models was associated with the clonal group case fatality in human infections. These results may be used in the risk stratification of CRAB strains.

## Supplementary Materials

Supplementary materials can be found at https://www.mdpi.com/2076-2607/8/6/847/s1. Supplementary Table S1: Minimum Inhibitory Concentration (MIC) (mg/L) of isolates. Supplementary Table S2: Results of in vitro and in vivo fitness tests.
